# Current News

**Published:** 1904-05

**Authors:** 


### Current News.

### PENNSYLVANIA STATE BOARD OF DENTAL EXAMINERS.

The Board of Dental Examiners of Pennsylvania will conduct examinations simultaneously in Philadelphia and Pittsburg, June 8 to 11, 1904. Applicants for examination and license must address the Hon. C. N. Schaeffer, Secretary Dental Council, Harrisburg, Pa., for papers or any further information.

---

### NATIONAL ASSOCIATION OF DENTAL FACULTIES.

The next annual meeting of the National Association of Dental Faculties will convene at 10 a. m., June 9, 1904, in Washington, D. C. The Executive Committee will be in session the afternoon of June 8 to consider such matters as may be brought before it. Arrangements are being made with the railroads for one and one-third fare on the certificate plan. The hotel as headquarters, together with the railroad rates, will be announced later by circular letters to the colleges.

H. B. Tileston, *Chairman.*
S. W. Foster, *Secretary.*

---

### SOUTH DAKOTA STATE BOARD OF DENTAL EXAMINERS.

The South Dakota State Board of Dental Examiners will hold its next regular session for the examination of applicants for license, at Aberdeen, South Dakota, June 9, beginning at 1.30 p.m. All applicants will be required to insert at least two gold fillings, and such other work as the Board may require. Besides the regular operating instruments each candidate is required to bring a bridge of not less than four teeth, including one Richmond crown and one molar shell crown, invested ready for soldering. Application must be made to the secretary at least one week before examination takes place.

G. W. Collins,
*Secretary.*

---

### PROGRAMME OF THE AMERICAN MEDICAL ASSOCIATION, SECTION ON STOMATOLOGY.

(Atlantic City, N. J., June 7 to 10, 1904.)

1. "The Value of Symmetry in the Development of Professional Character and Education" (Chairman's Address), George F. Eames, Boston, Mass.
2. "The Evolution of Standards in Dental Education," Charles Chittenden, Madison, Wis.
3. "Phases of Dental Education," A. E. Baldwin, Chicago, Ill.
4. "Dental Education: A Retrospective and Prospective View," John S. Marshall San Francisco, Cal.
5. "Neoplasm of the Tooth-Pulp," Vida A. Latham, Chicago, Ill.
6. "Vital Principles in Adult Pulp," R. R. Andrews, Cambridge, Mass.
7. "Degeneration of the Tooth-Pulp," Eugene S. Talbot, Chicago, Ill.
8. "The Pulp," Jos. Arkövy, Budapest, Hungary.
9. "A System for Surgical Treatment of Harelip, Cleft Palate, and Facial Deformities and Post-Operative Speech Education," George V. I. Brown, Milwaukee, Wis.
10. "Multiple Fracture of Lower Jaw complicated by Simultaneous Fracture of the Upper Jaw," Thomas L. Gilmer, Chicago, Ill.
11. "Impacted Teeth: Their Diagnosis, Liberation, and Extraction," Matthew H. Cryer, Philadelphia, Pa.
12. "Ankylosis of the Jaw," G. Lenox Curtis, New York City, N. Y.
13. "Necrosis of the Bones of the Face," Stewart L. McCurdy, Pittsburg, Pa.

14. "Treatment of Pathological Irregularities of the Teeth," M. H. Fletcher, Cincinnati, Ohio.

15. "Report of a Case of Vincent's Angina and Stomatitis, with Photographs," George C. Crandall, St. Louis, Mo.

16. "Oral Infection and Sterilization," M. L. Rhein, New York City, N. Y.

17. "Concerning Changes in the Salivary Secretions as affected by Systemic Disease," Heinrich Stern, New York City, N. Y.; William Lederer, New York City, N. Y.

18. "Prophylaxis in Relation to Tooth Environment and to the Prophylactic Value of Materials employed," Chas. F. Allan, Newburgh, N. Y.

19. "The Physician's Duty to the Child from a Dental Standpoint," Alice M. Steeves, Boston, Mass.

20. "Ethics," Adelbert H. Peck, Chicago, Ill.

DR. GEORGE F. EAMES, *Chairman.*
EUGENE S. TALBOT, *Secretary.*

---

### NORTHERN INDIANA DENTAL SOCIETY.

THE sixteenth annual meeting of the Northern Indiana Dental Society will be held in Huntington, Ind., on October 4 and 5, 1904.

Arrangements are being made to make this the greatest convention ever held in Northern Indiana. Already some of the best talent in the country has been secured.

OTTO U. KING,
*Secretary.*

---

### DENTAL COMMISSIONERS OF CONNECTICUT.

THE Dental Commissioners of Connecticut hereby give notice that they will meet at Hartford on May 14, as prescribed by law, and will adjourn to July for the summer examinations, so as to enable those students who do not finish their college or other educational course until June an opportunity to secure a license to practise without the long delay now made necessary because of being required to wait until November.

Hereafter the November examinations will be dispensed with until further notice.

Examinations to secure a license to practise dentistry in Connecticut will be held July 14, 15, and 16, 1904, at Hartford, Conn.

Full particulars will be published in the dental journals, or may be secured of the Recorder.

By direction of the Dental Commissioners.

J. TENNEY BARKER,
*Recorder.*

WALLINGFORD, CONN.

---

### CALIFORNIA STATE DENTAL ASSOCIATION.

THE Joint Clinic of the California State Dental Association and the Alumni Association, Dental Department, University of California, will be held May 16 to 19, 1904, in San Francisco.

Dr. Hart J. Goslee, of Chicago, will give a series of clinics on Porcelain, Dr. Henry A. Baker, of Boston, will give a series of clinics on Orthodontia and the Baker Anchorage, and a large local clinical programme is being arranged.

All the leading manufacturers have signified their intention of making an exhibit of their products, and the local dealers will also be represented.

The session is expected to surpass any previously held in this State.

GUY S. MILLBERRY,
*Secretary.*

---

### MINNESOTA STATE BOARD OF DENTAL EXAMINERS.

THE Minnesota State Board of Dental Examiners will hold a special meeting for the purpose of examining applicants for license, June 13, 14, and 15, 1904.

No applications received after 12 M. June 13. Meeting held at the Dental Department of the State University at Minneapolis.

C. H. ROBINSON,
*Secretary.*

WABASHA, MINN.

